# First person – Samia Pratt

**DOI:** 10.1242/dmm.052423

**Published:** 2025-06-02

## Abstract

First Person is a series of interviews with the first authors of a selection of papers published in Disease Models & Mechanisms, helping researchers promote themselves alongside their papers. Samia Pratt is first author on ‘ Evaluating the feasibility of gene replacement strategies to treat *MTRFR* deficiency’, published in DMM. Samia is a PhD student in the lab of Robert Burgess at The Jackson Laboratory, Bar Harbor, ME, USA, investigating potential gene therapeutics for rare inherited mitochondrial diseases and peripheral neuropathies.


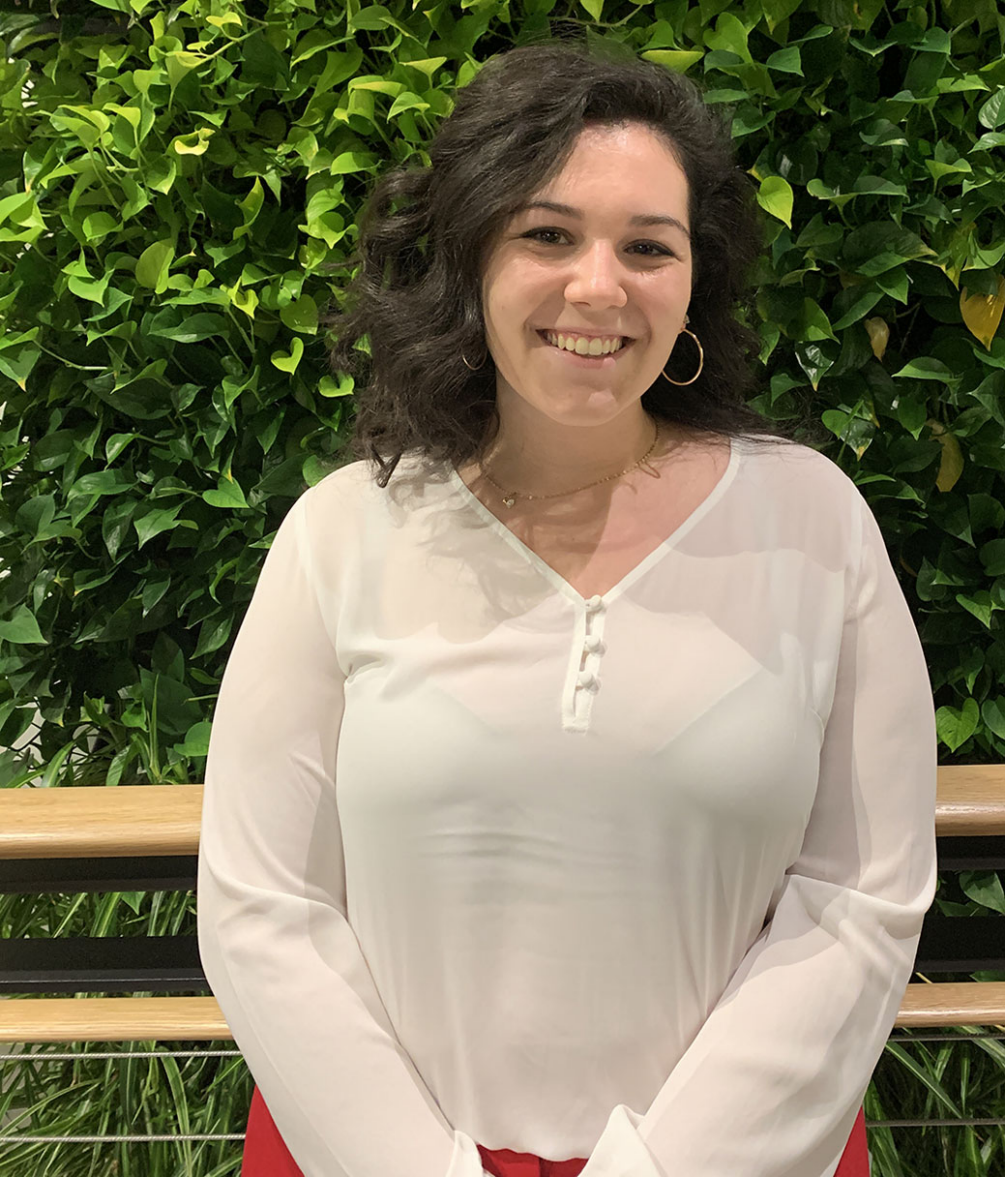


**Samia Pratt**

**Who or what inspired you to become a scientist?**

I was inspired to become a scientist at a very young age. I grew up in Rhode Island and spent a lot of my childhood on the beach. I was always curious about how animals were similar and different and what everything was made of. I had a loved one who was diagnosed with a neurodegenerative disease when I was young, and I became very inspired by disease mechanisms and the pathology that was driving them, what made some of us more susceptible to these diseases and why was it happening. I luckily had a great biology teacher in high school and attended an excellent summer program at the Shoals Marine Laboratory, and I fell even more in love with biology and trying to better understand what was happening in certain diseases at a cellular level.

**What is the main question or challenge in disease biology you are addressing in this paper? How did you go about investigating your question or challenge?**

The main questions we're addressing in this paper is whether gene replacement strategies may be a good treatment for *MTRFR* deficiency. MTRFR is a small protein that resolves stalled ribosomes in the mitochondria during mitochondrial translation and is very important for proper mitochondrial and overall cellular health. Patients that present with homozygous pathogenic variants in *MTRFR* are usually diagnosed with Behr's syndrome and have peripheral and optic neuropathy, spastic paraplegia and, in some cases, intellectual disability. Unfortunately, there are currently no treatments for *MTRFR* deficiency, and, as post-mitotic cells seem to be most affected in this disease, we wanted to examine if gene replacement strategies would be effective. To address this question, we built both *in vivo* and *in vitro* systems of *MTRFR* deficiency. We saw that, in our cellular models, we were able to rescue cells with adeno-associated virus (AAV) *MTRFR* treatment. In our mouse model, we saw that transgenic expression of human *MTRFR* was able to rescue the phenotype of MTRFR endogenous knockout. With these models, we provide a multi-system approach and validation that gene replacement may be a viable treatment option for *MTRFR* deficiency, although more work is needed to better understand the mechanism behind this.

**How would you explain the main findings of your paper to non-scientific family and friends?**

In this paper, we are working towards better understanding the mechanism of a rare disease (Behr's syndrome) and identifying potential treatment options for patients. The subset of Behr's syndrome we're looking at is caused by mutations in the MTRFR protein and affects a small number of patients. These patients start experiencing symptoms very young, usually around 2-3 years of age, and have progressive blindness, loss of coordination, muscle stiffness and intellectual disability. There are currently no treatments for these patients. As this is a very rare disease, there was no proper animal model for us to study what occurs during this disease, so we worked to create a mouse model that would mimic patient symptoms and give us more insight into possible treatments. Using a combination of our mouse and cell models, we learned that a gene therapy treatment might be a suitable treatment option, with further testing necessary.

**What are the potential implications of these results for disease biology and the possible impact on patients?**

These results are very encouraging for patients. Together, our work validates that gene replacement strategies, such as AAV treatments, may be a suitable treatment option for patients with *MTRFR* deficiency. Although more work is needed to be done to ensure the safety of this and further investigate the disease mechanism driving pathology, our results provide a system that can positively impact patients with *MTRFR* deficiencies with no overt negative effects of transgenic overexpression of MTRFR.

**Figure:**
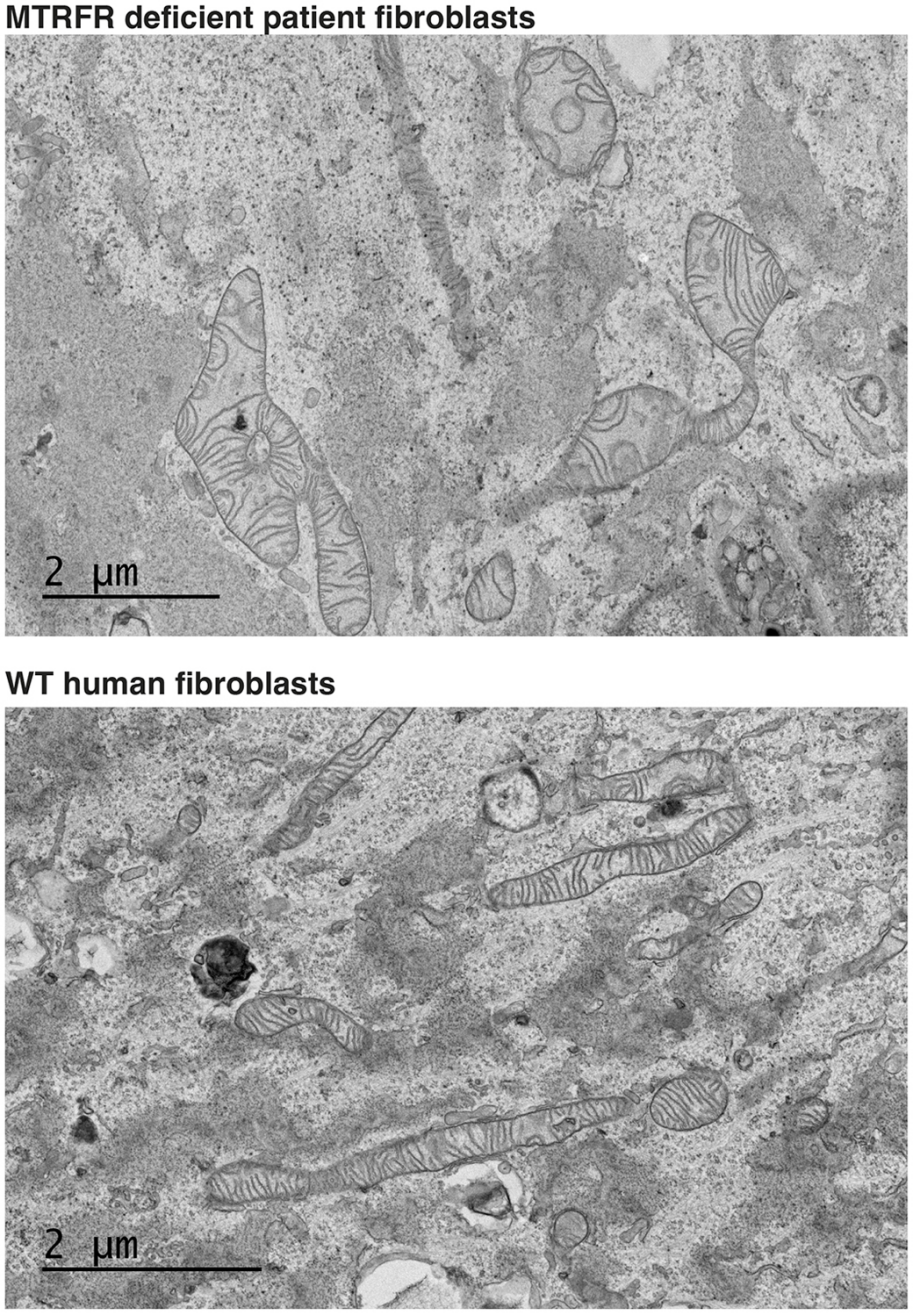
**Images of mitochondria in MTRFR-deficient patient fibroblasts and wild-type (WT) human fibroblasts to examine mitochondrial morphological changes with *MTRFR* deficiency.** Contributed by collaborators in the Battersby laboratory. Electron microscopy images were taken at 2500×.

**Why did you choose DMM for your paper?**

In this paper, we built new *in vivo* and *in vitro* models to better understand the pathology behind *MTRFR* deficiency and identify potential therapeutic targets. As this journal helps to highlight not only varying disease models but also research into rare diseases, we thought our paper would be a good fit for DMM.

[…] rare disease research is incredibly important, and basic science approaches of rare disease research can not only provide insight into that specific disease pathology but also into the pathology of other larger disease groups.

**Given your current role, what challenges do you face and what changes could improve the professional lives of other scientists in this role?**

As a graduate student studying a rare disease, it can be very difficult to get funding for these types of projects. Personally, I believe that rare disease research is incredibly important, and basic science approaches of rare disease research can not only provide insight into that specific disease pathology but also into the pathology of other larger disease groups. By understanding the mechanisms behind rare diseases, we are able to apply those findings to other diseases and better understand treatment options for the patients living with these diseases.

**What's next for you?**

I will be continuing to follow this project throughout the duration of my PhD and will follow up with a conditional knockout mouse model and an induced pluripotent stem cell approach to further understand cell-type specificity in *MTRFR* deficiency.

**Tell us something interesting about yourself that wouldn't be on your CV**

I come from a very musical family and am a huge fan of music and live music. I try to listen to as much diverse music as possible and am constantly working my way through various rankings of the top 100 albums of the past year and of all time.
